# Midwives’ experiences of using the Obstetric Norwegian Early Warning System (ONEWS): A national cross-sectional study

**DOI:** 10.18332/ejm/134510

**Published:** 2021-04-22

**Authors:** Line K. L. Fladeby, Marianne Raunedokken, Hannah E. Fonkalsrud, Dorte Hvidtjørn, Mirjam Lukasse

**Affiliations:** 1 Faculty of Health and Social Sciences, University of South-Eastern Norway, Kongsberg, Norway; 2 Department of Obstetrics, Sykehuset Telemark Hospital, Skien, Norway; 3 Department of Obstetrics, Sykehuset i Vestfold Hospital, Tønsberg, Norway; 4 Department of Clinical Research, University of Southern Denmark, Odense, Denmark; 5 Faculty of Health Sciences, Oslo Metropolitan University, Oslo, Norway

**Keywords:** maternal observations, Obstetric Early Warning System, Obstetric Norwegian Early Warning System

## Abstract

**INTRODUCTION:**

Increasing numbers of maternity units are implementing routine and standardized monitoring of all women using a form of Early Warning Score System with the aim to early detect women at risk of developing critical illness or a deterioration of their condition. The implementation in Norway is relatively new. This study aimed to describe Norwegian midwives’ experiences with the Obstetric Norwegian Early Warning System (ONEWS).

**METHODS:**

We performed a cross-sectional study based on an electronic questionnaire, sent to heads of midwifery at all maternity units in Norway for distribution to their clinically active midwives. Thirty-one of 48 units had implemented ONEWS for over a month. About 1020 midwives received the questionnaire, 232 (23%) responded.

**RESULTS:**

Of the participants, 217 (93.5%) reported receiving sufficient training and 230 (99.1%) reported using the same scoring system, including the same vital parameters measured. The criteria for use of ONEWS varied between units regarding inclusion criteria and frequency of scoring. A total of 214 (92.2%) midwives agreed that ONEWS has value in the surveillance of high-risk women, while 152 (65.5%) agreed that ONEWS contributes to medicalization of the care of low-risk women. Some 166 (71.6%) midwives reported that ONEWS was time consuming and 159 (68.5%) that the measures need to be better adapted to childbearing women.

**CONCLUSIONS:**

Maternity units in Norway implementing ONEWS use an almost identical scoring system but varying criteria for whom to score and how often. Midwives considered ONEWS particularly suited for high-risk women and not for low-risk childbearing women.

## INTRODUCTION

In Norway, the maternal mortality rate is among the lowest in the world and has remained stable over the last 30 years. In 2015, this rate was 5 per 100000 births^1^. However, nine times as many women who die, develop serious illness, most often haemorrhages, infections, hypertensive disorder or thrombosis^2,3^. Improved maternity care can prevent not only mortality but also morbidity. This assertion is supported by the report ‘Saving lives, Improving Mothers Care’ from the UK and Ireland in 2016 as well as by the WHO, both of which claim that qualified help, simple observations and quick action have the potential to prevent severe illness, adverse outcomes and mortality^2,4^.

One way to ensure adequate observations and appropriate action is by using a standardized system^5^. During the last decade, a number of standardized Obstetric Early Warning Systems (OEWS) have been developed internationally^6,7^. They consist of a systematic and objective scoring tool based on vital parameters such as respiratory rate (RR), oxygen saturation (SpO2), temperature, heart rate (HR), blood pressure (BP) and state of consciousness. The parameters provide a total score which is color-coded and triggers further interventions such as increased monitoring and alerting a doctor if necessary^6,7^. The Obstetric Early Warning System (OEWS) is for women during pregnancy, perinatal and postnatal care, from confirmation of pregnancy to six weeks postpartum, but not during active delivery. This is because the delivery often causes parameters to rise as a response to natural processes such as pain^8^.

The Obstetric Norwegian Early Warning System (ONEWS)^9^ is based on the Irish version, Irish Maternity Early Warning System (IMEWS), which was introduced in 2013 as the first national guideline of its kind^3^. ONEWS was included in the national guidelines for obstetrics in February 2020^9^. Prior to this, ONEWS was presented and discussed at seminars open for both doctors and midwives where obstetricians from Ireland explained the score system and shared their experience^10^. A substantial number of maternity units, in particularly high-risk units, started using ONEWS prior to the publication of the national guidelines, which could have resulted in differences in practice. Variation across units in uptake and use is also known from other countries, such as the UK^11^.

International studies on the implementation of OEWS reveal a number of barriers, among them the midwives’ concern for unnecessary intrusion on the patient group, increased workload and lack of clarity regarding its purpose^6-8,11-13^. The organization and culture of maternity care and the role and autonomy of midwives varies considerably between different countries, even within Europe^14^. Thus, the results of these studies are not necessarily applicable to the Norwegian setting.

So far, there is no research published on the use of ONEWS in Norway. The aim of our study was twofold. Firstly, to describe the uptake and criteria for use of ONEWS in Norway by clinically active midwives with experience of using ONEWS. Secondly, to describe those same midwives’ perception of the use of ONEWS.

## METHODS

This is a cross-sectional study based on data from electronic questionnaires.

### Recruitment of the sample

All existing maternity units in Norway were identified at the webpage Helsenorge^15^. Contact was established with heads of midwifery/leading midwives of all units, informing them about the study and asking for their permission and help to distribute the questionnaire to all clinically active midwives within their unit. Response from the contacted midwives revealed that by January 2020, the start of this study, 34 of the existing 48 maternity units in Norway had started using ONEWS. These included obstetric units (high-risk), maternity wards (lower risk), and midwife-led units (low-risk)^15^. Three units had used ONEWS less than a month and were therefor excluded from the study. Gynaecological wards that admitted patients for treatment during pregnancy and/ or the postnatal period were excluded from our study.

An anonymous questionnaire was prepared through the *nettskjema.no* online service (University in Oslo 2018). The introduction to the questionnaire described the study’s objective and its measures to protect privacy. The respondents consented to participation by responding to the questionnaire. No responses or IP addresses could be traced back to individual respondents, and the respondents were therefore able to withdraw from participation.

Permission to distribute the questionnaires was obtained from each head of midwifery and/or leading midwife by the 8 January 2020. These midwives were asked to distribute the questionnaire to all clinically active midwives in their maternity units via work email. After one week, a reminder was sent to the respective heads of midwifery/leading midwives. On 28 January, information about the study was posted on a closed Facebook group for Norwegian midwives. The post asked midwives to check if they had received a link to the questionnaire at work and asked them to complete the questionnaire if they had not yet done so. A second identical reminder was posted on 3 February. No link to the survey was posted on this Facebook page. The survey was closed to participation on 9 February. With the information from the contact midwives at the units, we estimated that a total of 1020 clinically active midwives received the questionnaire, in 29 of the 31 maternity units. Altogether 232 (23%) midwives completed the questionnaire.

### The Norwegian obstetric care setting

In Norway, maternity units are divided into three levels: obstetric clinics (high-risk), maternity wards (lower risk), and midwife-led units (low-risk)^16^. This three-tier structure presumes selection of women to the appropriate level of care depending on their risk level^17^. Furthermore, the midwife has the primary responsibility for women in pregnancy, perinatal and postnatal care, and uncomplicated childbirth that does not require the involvement of a doctor^16^. Midwives are responsible for recording vital parameters and contacting a doctor when these deviate from the normal.

### The survey

No validated questionnaires assessing the uptake and use of EWS are available. Thus, we based our questionnaire on evidence from the literature on obstetric Early Warning Systems and used questions from previously published studies on this topic^8,11,12,18,19^. The questions on which vital signs were registered, which parameters were lacking, training in use of EWS, time usage, criteria for use, and appropriateness for childbearing women were those used in the study by Carlstein et al.^8^ who referred to the studies by Isaac et al.^12^ and Bick et al.^11^ and translated them into the Scandinavian languages. The questions on medicalization of childbirth, threat to the midwife’s clinical judgment, and intrusiveness, are based on the qualitative findings by Martin^19^ and those of Jeffery et al.^18^. The questions on increased safety and communication value were derived from the study by Mackintosh et al.^13^. Once the authors considered the questionnaire complete, a face-value pilot test of the questionnaire was undertaken, with seven final year midwifery Master’s students not involved in the development of the study and two clinically active midwives, all with experience of ONEWS. Some minor amendments and additions were made as a result of this. Subsequently, the electronic version was tested for programming issues and completeness of data.

The final questionnaire consisted of three parts with a total of 22 questions (Supplementary file). The questionnaire was programmed such that each question had to be answered to be able to move to the next question, but with ‘don't know’ and ‘no’ as options. The first part included demographic variables such as age, education level, years of midwifery experience, geographical location (health authority) and type (level of risk) and size of the maternity unit (number of births per year). The second part focused on the length of time that ONEWS had been used and the type and quality of teaching/training that had been provided before its implementation. This part also included questions on the criteria for using ONEWS, i.e. which women and when, and the respondent’s opinion regarding the vital parameters of the monitoring system. Participants were also asked which other measures they felt should be included in ONEWS.

The third part of the survey focused on the midwives’ clinical experience with ONEWS, where the midwives were asked to agree or disagree with a number of statements. The response alternatives were given on a 5-point Likert scale ranging: 1=fully agree to 5=fully disagree. The final question in part three was open-ended to potentially elicit other information regarding the midwives’ experiences with using ONEWS.

### Data analysis

Descriptive analyses were undertaken to describe the respondents, presented in frequencies and percentages (Table 1). Based on the literature we hypothesized that the results could differ according to the level of risk midwives commonly worked with, their experience as a midwife and their experience with using ONEWS^8,11,13,18^. Chi-squared test was used for the bivariate analyses that investigated criteria for use of ONEWS and midwives’ opinions on ONEWS by type of maternity unit (Tables 2 to 4). For these analyses, the maternity units were divided into high-risk (obstetric units), lower risk, and low-risk units (i.e. all the other units, such as maternity ward, midwife led units). Scaled response alternatives for the opinion statements were dichotomized. The answering options ‘totally agree’ and ‘partially agree’ were categorized as ‘agree’, the options ‘neither agree nor disagree’, ‘partially disagree’ and ‘totally disagree’ as ‘disagree’. The results for ‘agree’ are presented in the tables. Chi-squared tests were also performed to assess if length of experience as a midwife and time of experience with ONEWS was associated with the reported criteria for use of ONEWS, measures suggested to be included in ONEWS and opinion statements on ONEWS, are presented in the text. For this analysis, years of midwifery experience were dichotomized into <10 years and ≥10 years. Experience with using ONEWS was dichotomized into ≤1 year and >1 year. Due to the programming of the questionnaire, we had no missing data. The statistics application IBM Statistical Package for Social Science (SPSS) version 26 was used to analyze the data. Comments from the free text opportunities are used to illustrate the qualitative findings.

**Table 1:** Demographic characteristics of participating midwives (N=232)

*Characteristics*	*n (%)*
**Age** (years)	
<35	54 (23.3)
35–46	88 (37.9)
47–58	62 (26.7)
>58	28 (12.1)
**Experience as a midwife** (years)	
0–9	100 (43.1)
10–25	88 (37.9)
>25	44 (19.0)
**Education level**	
Diploma in midwifery	179 (77.2)
Master’s in midwifery	53 (22.8)
**Health authority** (region)	
West	27 (11.6)
Central	22 (9.5)
North	22 (9.5)
South-Eastern	161 (69.4)
**Type of maternity unit**	
Obstetric unit (high-risk level)	129 (55.6)
Maternity ward (lower risk level)	95 (40.9)
Midwife-led unit (low-risk level)	8 (3.5)
**Size of unit by number of births per year**	
<500	37 (15.9)
500–999	23 (9.9)
1000–1999	110 (47.4)
2000–3000	17 (7.3)
>3000	45 (19.5)
**Length of time ONEWS used at participant’s unit**	
1–5 months	44 (19.0)
6–12 months	72 (31.0)
1–2 years	98 (42.2)
>2 years	17 (7.3)
Do not know	1 (0.5)
**Length of experience with ONEWS**	
One year or less	112 (48.3)
More than one year	100 (43.1)
Not sure	20 (8.6)
**Type of education prior to using ONEWS**	
Day course at own unit	183 (78.9)
Other (colleague, online course)	46 (19.8)
No education at all	3 (1.3)

**Table 2:** Vital parameters reported to be included in ONEWS (N=232)

*Parameters*	*Low-risk and lower risk units*	*High-risk unit*	*Total*
	*(n=103) n (%)*	*(n=129) n (%)*	*n (%)*
Respiration frequency	102 (99.0)	128 (99.2)	230 (99.1)
Oxygen saturation	102 (99.0)	127 (98.4)	229 (98.7)
Temperature	103 (100)	128 (99.2)	231 (99.6)
Pulse	103 (100)	128 (99.2)	231 (99.6)
Blood pressure	103 (100)	128 (99.2)	231 (99.6)
Level of consciousness	102 (99.0)	126 (97.7)	228 (98.3)
Total yellow score	99 (96.1)	124 (96.1)	223 (96.1)
Total red score	100 (97.1)	124 (96.1)	224 (96.6)
Urine (protein, glucose, other)	64 (62.1)	71 (55.0)	135 (58.2)
Pain score 0–10	55 (53.4)	71 (55.0)	126 (54.3)
Doctor contacted	85 (82.5)	107 (82.5)	192 (82.8)
Other	7 (6.8)	4 (3.1)	11 (4.7)

## RESULTS

**Figure 1:**
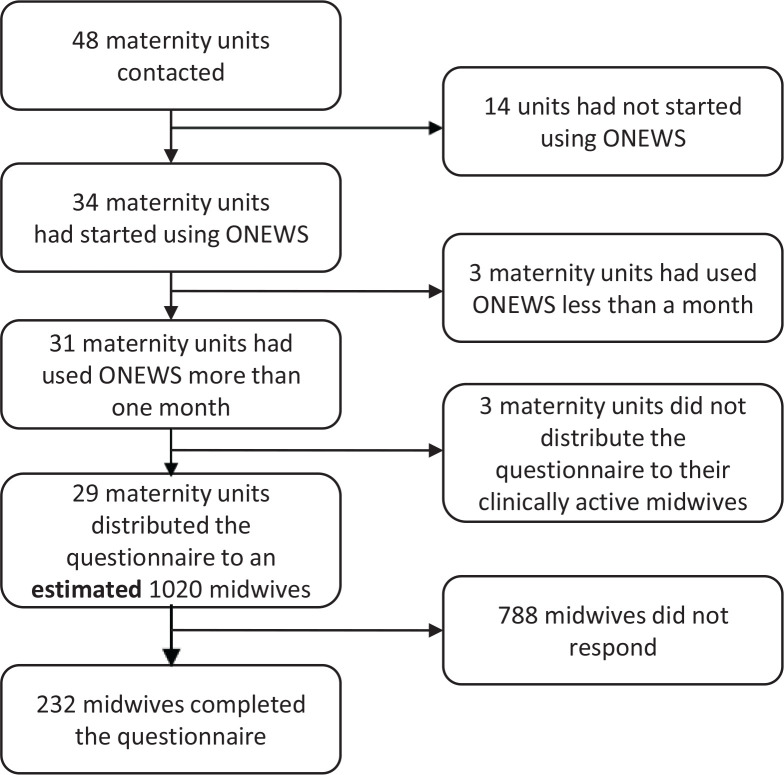
Sample flowchart

The selection of the sample is shown in Figure 1. Of the 232 participating midwives, 44 (19.0%) reported that ONEWS had been used ≤5 months at their unit while 100 (43.1%) midwives had personal experience using ONEWS for >1 year (Table 1). Most midwives had received training in the use of ONEWS at a one-day course at their own unit (n=183; 78.9%) and 217 (93.5%) felt that this training was sufficient. Altogether 212 (91.4%) midwives answered that they did not use ONEWS for monitoring women during active birth.

When asked how frequently the women were scored with ONEWS (multiple answers possible), the majority answered ‘once per day’ (n=76; 32.8%) or ‘twice per day’ (n=108; 46.6%). The free-text comments explained some of the background for varying frequencies: *‘no-risk women once per day; high-risk women morning and evening; and otherwise on indication.’*

The recommended vital parameters respiration, oxygen saturation, temperature, blood pressure, pulse and level of consciousness were included in the ONEWS scoring form used by almost all the midwives (Table 2). Midwives working at a high-risk unit reported monitoring all women with ONEWS within two to three hours postpartum more often than midwives working at a lower risk or low-risk unit (71.3 % vs 56.3%, p=0.018) (Table 3). Midwives working at a high-risk unit used ONEWS more often prior to early discharge (14.7%) compared to midwives working at lower level risk (4.9%) (p=0.014) (Table 3).

**Table 3:** Midwives’ reports of criteria for use of ONEWS by level of obstetric risk unit* (N=232)

	*Low-risk and lower risk units*	*High-risk unit*	*Total*
	*(n=103) n (%)*	*(n=129) n (%)*	*n (%)*
**Criteria for monitoring with ONEWS during pregnancy**			
All women in outpatient consultations	29 (28.2)	45 (34.9)	74 (31.9)
Only women with high-risk pregnancy in outpatient consultation	4 (3.9)	3 (2.3)	7 (3.0)
All women admitted until discharge or birth	65 (63.1)	79 (61.2)	144 (62.1)
All women admitted for observation, discontinued when the parameters are normal	23 (22.3)	19 (14.7)	42 (18.1)
Only women with high-risk pregnancy admitted for observation, until discharge or birth	15 (14.6)	19 (14.7)	34 (14.7)
Don’t know	3 (2.9)	5 (3.9)	8 (3.4)
Other	6 (5.8)	11 (8.5)	17 (7.3)
**Criteria for monitoring with ONEWS after vaginal birth**			
All women within 2–3 hours postpartum	58 (56.3)	97 (71.3)	150 (64.7)
All women within 2–3 hours postpartum, further monitoring discontinued when parameters are normal	24 (23.3)	27 (20.9)	51 (22.0)
Only women classified as high-risk, within 2–3 hours postpartum	11 (10.7)	5 (3.9)	16 (6.9)
Don’t know	1 (1.0)	0 (0.0)	1 (0.4)
Other	11 (10.7)	12 (9.3)	23 (9.9)
**Criteria for monitoring with ONEWS after a caesarean section**			
All women after a CS and until discharge	77 (74.8)	102 (79.1)	179 (77.2)
All women after a CS, further monitoring discontinued when parameters are normal	23 (22.3)	24 (18.6)	47 (20.3)
Don’t know	1 (1.0)	1 (1.0)	2 (1.0)
Other	3 (2.9)	2 (1.6)	5 (2.2)
**Criteria for monitoring with ONEWS in the postnatal ward**			
All women with early discharge, 4–24 hours after birth	5 (4.9)	19 (14.7)	24 (10.3)
All women in the postnatal ward, until discharge	55 (53.4)	64 (49.6)	119 (51.3)
Only women in the postnatal ward who are classified as high-risk	18 (17.5)	31 (24.0)	49 (21.1)
Only women in the postnatal ward who are classified as high-risk, discontinued when parameters normal	21 (20.5)	25 (19.4)	46 (21.1)
Don’t know	2 (1.9)	7 (5.4)	9 (3.9)
Other	14 (13.6)	14 (10.9)	28 (12.1)
**Criteria for monitoring with ONEWS upon readmission**			
All women readmitted within 6 weeks postpartum until discharge	55 (53.4)	57 (44.2)	112 (48.3)
All women readmitted within 6 weeks postpartum, discontinued when parameters normal	6 (5.8)	13 (10.1)	19 (8.2)
Women only on indication	13 (12.6)	17 (13.2)	19 (8.2)
Don’t know	26 (25.5)	35 (27.1)	61 (26.3)
Other	4 (3.9)	9 (7.0)	13 (5.6)

* More than one answer possible, so row percentages.

Checking the uterus (n=106; 45.7%) and vaginal bleeding (n=73; 31.5%) were regarded as the most relevant parameters to be included in addition to the current parameters.

Significantly fewer midwives employed in high-risk units reported seeing a clear purpose with the use of ONEWS compared to those working in lower risk or low-risk units (65.9% vs 79.6%, p=0.021) (Table 4). In all, 96 (41.4%) midwives considered the vital thresholds used in ONEWS

**Table 4:** Number and proportion of midwives who agreed with the statements below by type of maternity unit in which they work (N=232)

	*Low-risk and lower risk units*	*High-risk unit*	*Total*
	*(n=103) n (%)*	*(n=129) n (%)*	*n (%)*
*‘I see a clear purpose with the use of ONEWS.’*	82 (79.6)	85 (65.9)	167 (72.0)
*‘I believe that ONEWS helps improve our knowledge about monitoring of vital parameters.’*	75 (72.8)	89 (69.0)	164 (70.7)
*‘In my experience, ONEWS is often deprioritized in favor of other tasks that are considered more clinically relevant.’*	31 (30.1)	42 (32.6)	73 (31.5)
*‘I believe that monitoring with ONEWS needs to be better adapted to each individual woman in the unit.’*	66 (64.1)	93 (72.1)	159 (68.5)
*‘I believe that ONEWS helps improve patient safety.’*	79 (76.7)	80 (62.0)	159 (68.5)
*‘I feel that monitoring with ONEWS is time-consuming.’*	71 (68.9)	95 (73.6)	166 (71.6)
*‘I believe that ONEWS leads to better procedures for systematic monitoring.’*	81 (78.6)	97 (75.2)	178 (76.7)
*‘I believe that monitoring with ONEWS is intrusive for the women, because of all the interruptions it involves.’*	41 (39.8)	64 (49.6)	105 (45.3)
*‘I feel that it is challenging to defend monitoring with ONEWS to women who are not at any risk.’*	55 (53.4)	77 (59.7)	132 (56.9)
*‘In my experience, ONEWS helps to better reveal early signs of illness.’*	59 (57.3)	58 (45.0)	117 (50.4)
*‘I consider monitoring with ONEWS to be important when the woman is classified high-risk or shows signs of illness.’*	98 (95.1)	116 (89.9)	214 (92.2)
*‘I find that ONEWS causes an increased medicalization of normal pregnancy, perinatal and postnatal care practices.’*	64 (62.1)	88 (68.2)	152 (65.5)
*‘In my contacts with the obstetrician, I feel that it is easier to be heard if I can refer to an ONEWS score.’*	59 (57.3)	77 (59.7)	136 (58.6)
*‘I believe that ONEWS is a threat to the midwife's clinical judgement.’*	35 (34.0)	52 (40.3)	87 (37.5)

not well adapted to childbearing women. Significantly fewer midwives believed that the threshold values were adapted to childbearing women after having worked with ONEWS for more than one year (n=42; 35.0%), when compared to those who had worked with ONEWS for less than one year (n=54; 48.2%) (p=0.041). The threshold values that the midwives felt were not well adapted included pulse, temperature and respiratory rate, as illustrated by the comments: *‘pulse over 90 and temp above 37.4 are often seen in women postpartum without this being an indication of illness’, and ‘respiratory rate is a little too “strict” – it's often higher in the first hour after birth. They can often have an RR of 20–22.’*

One of the midwives wrote that: *‘pregnant women early in the gestation period do not necessarily match women who are admitted for pregnancy observation before birth or induction, or women who are postpartum’*. Another midwife commented that: *‘the evidence base is not yet strong enough to draw conclusions about what should be considered normal variation in pregnancy, the delivery and the postnatal period’*.

A total of 152 (65.5%) midwives were of the opinion that monitoring with ONEWS represented a medicalization of maternity care (Table 5). Most midwives felt that ONEWS was time-consuming (n=166; 71.6%). A total of 159 (68.5%) midwives agreed that ONEWS helped improve patient safety, with significantly more midwives who worked in low-risk and lower risk units compared to those working at a high-risk unit (76.7% vs 62.0%, p=0.017) (Table 5). Nearly all midwives agreed that monitoring with ONEWS was important for women who were at risk or showed signs of illness (n=214; 92.2%).

## DISCUSSION

The midwives in our study reported sufficient training in ONEWS prior to implementation and using a largely identical scoring system. Midwives working at a high-risk unit reported using routine ONEWS for all women postpartum and prior to early discharge significantly more often than midwives working at low-risk and lower risk units. Midwives who had used ONEWS for over a year were significantly more likely to consider threshold values not well adapted to childbearing women compared to midwives with less experience with ONEWS. Irrespective of place of work, midwifery and/or ONEWS experience, midwives considered ONEWS time consuming, increasing medicalization of childbirth, important for high-risk women and increasing patient safety.

In contrast to the study by Martin^19^, the midwives in our study reported sufficient training prior to implementation of ONEWS. Our findings on the uptake of the different aspects of the ONEWS are very similar to those found by Bick et al.^11^ and Isaac et al.^12^. Similarly, we found almost 100% registration of respiration, pulse, temperature, blood pressure and oxygen saturation^11,12^. In agreement with their studies, urine testing for protein was reported by <60%, while registering level of consciousness was reported more frequently in our study (98.3%) than in the study by Bick et al.^11^ (78%). During the introduction of ONEWS, a national group of obstetricians and midwives was established to write the national guidelines for its use and inclusion in the national guidelines for obstetric care in Norway^9,10^. They organized seminars, meetings and workshops^10^. Thus, it was expected that the scoring system was similar in all units.

In contrast to the intention of ONEWS as a screening instrument for all women^6^, the midwives in our study reported more frequent use of ONEWS for high-risk women. In addition, midwives did view ONEWS as a good tool to monitor in particular high-risk women. Selecting women to the appropriate level of care, in relation to their medical and obstetric level of risk, has a long tradition in Norway^16^. As the distance to the nearest obstetric unit may be long, midwives in Norway are used to identify childbearing women’s level of risk at every stage of the pregnancy, labor and postpartum period^17^. Even in obstetric units with a mixture of risk, laboring women are selected for care appropriate to their level of obstetric and medical risk, in order to avoid unnecessary interventions^20^. This may explain why Norwegian midwives view and apply ONEWS as a monitoring tool primarily for high-risk women. This approach of adapting the level of care to the level of risk is in line with WHO guidelines for maternity care^21^.

ONEWS, IMEWS and similar assessment instruments have been adapted from scoring systems in the field of medicine that deals with sick people only, often critically ill patients^22^. As most births take place in hospital and the majority of childbearing women are healthy, with uncomplicated pregnancies, births, and pregnancies, many ‘obstetric patients’ are not sick but going through a normal process. This normal process, however, can imply a major physical and emotional challenge and effort to women. Physiology in pregnancy and labor is different from the non-pregnant state. Thus, if a monitoring system is used, it needs to allow for this ‘other normal’. The midwives in our study, particularly those with the most experience with ONEWS, did not consider the vital parameters well adapted to childbearing women. This concurs with the findings from the Scandinavian survey by Carlstein et al.^8^, where only a third of the midwives felt that the threshold values were well adapted. In contrast, in the studies by Bick et al.^11^ and Isaacs et al.^12^ the majority of respondents believed the values to be appropriate. However, in the study by Bick et al.^11^, only heads of midwifery were included. These respondents may not have used the scoring system themselves and may have focused mainly on risk reduction. In the study by Isaacs et al.^12^ senior obstetric anaesthesiologist, caring predominantly for obstetric women in critical care, were the main respondents. The midwives in our study, in agreement with other studies, mentioned that specific postpartum measures such as checking the uterus and vaginal bleeding are essential for monitoring postpartum women, and lacking in ONEWS^11,12^.

Midwives in our study found ONEWS time consuming. When staff and equipment are available, it is easy to introduce new routines, surveillance, even screening, without a proper assessment of its added value^13,23^. Ideally, prior to the introduction of a screening tool/system, high quality studies are performed to assess the sensitivity and specificity, as well as the positive and negative predictability of the screening tool to identify the predefined treatable condition in the target population^24^; in this case, the early detection of critical illness in all childbearing women. In addition, it is recommended practice to investigate the costs of the implementation of a screening tool/system, including equipment and time spent by staff^24^. There are a number of validation studies on Obstetric Early Warning Systems (OEWS) in obstetrics^7,25-27^. However, their evidence so far is limited, due to study design^27^, population included^26^, location of study reducing generalizability^25^ and a different purpose than early detection of critical illness^7,26^. In their systematic review Umar et al.^5^ conclude that EWS tools are highly accurate in their predictions of maternal mortality among critically ill obstetric patients, but that there is little evidence of their effectiveness for preventing maternal mortality in general, and that more robust studies are needed. None of the validation studies has taken cost and workload into account. However, several studies have reported midwives’ views on using EWS and mention the challenge of increased workload^8,13,19^. The seven month long ethnographic study by Macintosh et al.^13^, which included 120 hours of observation and 45 interviews at two UK hospitals, reported that midwives questioned the value of an extra chart resulting in increased workload.

Our findings show that two-thirds of the midwives believed that ONEWS turns normal childbirth into a medical condition. This is in agreement with the midwives in the study by Macintosh et al.^13^ who perceived the repeated scoring as a medicalization of the normal conditions in maternity care. In the same study, however, anaesthesiology personnel considered scoring of all women was justified in order to find the one woman who develops serious illness^13^. While, the Scandinavian midwives in the study by Carlstein et al.^8^ stated that vital parameters, influenced by normal labor, can be identified as abnormal values without any underlying pathology and consequently lead to unnecessary interventions.

### Strengths and limitations

Our study has a number of strengths as well as limitations. All four health authorities and nearly all maternity units that were using ONEWS distributed the questionnaire to their clinically active midwives. Our contact persons at each unit estimated the number of midwives they had sent the link of the questionnaire. Based on this estimate, the response rate was low. The distribution method was chosen to ensure that the midwives included were in fact users of ONEWS. However, clinical midwives may for various reasons not have accessed their work email during our short study period. In addition, we do not know for certain if, and how often, the contact midwives distributed the link to the midwives. If the low response rate is merely due to the short study period and method of data collection it may not have led to selection bias. In case of the most interested midwives responding, we are not certain how this may affect our findings. In any case, the low response rate gives reason for caution in generalizing the findings to all midwives in Norway.

The above notwithstanding, the respondents represented a wide range of age groups and years of service, as well as a large geographical area. The proportion of midwives in the different age groups was very similar to another national Norwegian study among clinically active midwives^28^. However, in our study we recruited fewer (about 10% less) midwives with 10 or more years of clinical experience compared to this previous national study^28^. The rate of participation was approximately equal between the maternity wards and the maternity clinics, but there were very few respondents from midwife-led units. The questionnaire used questions from other studies’ questionnaires, and some results were therefore comparable to those found in previous studies. Using an electronic questionnaire ensured minimal missing data from those who participated.

## CONCLUSIONS

ONEWS appears to be in the process of being implemented in all maternity units in Norway. Our study found that the same vital parameters and a similar scoring system was used across the units included in the study. There is a broad consensus by midwives that ONEWS plays an important role in the monitoring of women who are at an elevated risk of morbidity, but also that ONEWS causes an increased medicalization of normal maternity care practices. Seen in the light of the Norwegian setting with differentiated maternity care practice, which selects women to care relevant to their level of medical and obstetric risk, further research should focus on whether or not the early warning system is suitable for the low-risk and lower risk childbearing women.

## References

[1] Pregnancy-related mortality in Norway Svangerskapsrelatert dødelighet i Norge.

[2] Knight M, Nair M, Tuffnell D, Shakespeare J, Kenyon S, Kurinczuk J Saving Lives, Improving Mothers' Care-Surveillance of maternal deaths in the UK 2012-14 and lessons learned to inform maternity care from the UK and Ireland Confidential Enquiries into Maternal Deaths and Morbidity 2009-14.

[3] Department of Health (2019). Irish Maternity Early Warning System (IMEWS) V2.

[4] (2018). Individualized, supportive care key to positive childbirth experience, says WHO.

[5] Umar A, Ameh CA, Muriithi F, Mathai M (2019). Early warning systems in obstetrics: A systematic literature review. PLoS One.

[6] Swanton RDJ, Al-Rawi S, Wee MYK (2009). A national survey of obstetric early warning systems in the United Kingdom. Int J Obstet Anesth.

[7] Carle C, Alexander P, Columb M, Johal J (2013). Design and internal validation of an obstetric early warning score: secondary analysis of the Intensive Care National Audit and Research Centre Case Mix Programme database. Anaesthesia.

[8] Carlstein C, Helland E, Wildgaard K (2018). Obstetric early warning score in Scandinavia. A survey of midwives' use of systematic monitoring in parturients. Midwifery.

[9] Øverland EA, Ellingsen L, Heide HC, Aaby E, Einarson E, Nordhagen I (2020). ONEWS: Obstetric Norwegian Early Warning Score System.

[10] ONEWS erfaringskonferanse Kompetansebroen.

[11] Bick DE, Sandall J, Furuta M (2014). A national cross sectional survey of heads of midwifery services of uptake, benefits and barriers to use of obstetric early warning systems (EWS) by midwives. Midwifery.

[12] Isaacs RA, Wee MY, Bick DE (2014). A national survey of obstetric early warning systems in the United Kingdom: five years on. Anaesthesia.

[13] Mackintosh N, Watson K, Rance S, Sandall J (2014). Value of a modified early obstetric warning system (MEOWS) in managing maternal complications in the peripartum period: an ethnographic study. BMJ Qual Saf.

[14] Vermeulen J, Luyben A, O'Connell R, Gillen P, Escuriet R, Fleming V (2019). Failure or progress? The current state of the professionalisation of midwifery in Europe. Eur J Midwifery.

[15] Fødeplass og fødetilbud - slik får du det Helsenorge.

[16] Helsedirektoratet (2010). Et trygt fødetilbud: Kvalitetskrav til fødselsomsorgen.

[17] Regjeringen (2009). En gledelig begivenhet: Om en sammenhengende svangerskaps-, fødsels-og barselomsorg.

[18] Jeffery J, Hewison A, Goodwin L, Kenyon S (2017). Midwives' experiences of performing maternal observations and escalating concerns: a focus group study. BMC Pregnancy Childbirth.

[19] Martin RL (2015). Midwives’ experiences of using a modified early obstetric warning score (MEOWS): a grounded theory study. Evidence Based Midwifery.

[20] Bernitz S, Rolland R, Blix E, Jacobsen M, Sjøborg K, Øian P (2011). Is the operative delivery rate in low-risk women dependent on the level of birth care? A randomised controlled trial. BJOG.

[21] World Health Organization (2018). WHO recommendations: intrapartum care for a positive childbirth experience.

[22] Royal College of Physicians (2012). National Early Warning Score (NEWS): Standardising the assessment of acuteillness severity in the NHS. Report of a working party. Registered Charity No 210508.

[23] Friedman AM, Campbell ML, Kline CR, Wiesner S, D'Alton ME, Shields LE (2018). Implementing Obstetric Early Warning Systems. AJP Rep.

[24] Adriaensen WJ, Matheï C, Buntinx FJ, Arbyn M (2013). A framework provided an outline toward the proper evaluation of potential screening strategies. J Clin Epidemiol.

[25] Singh A, Guleria K, Vaid NB, Jain S (2016). Evaluation of maternal early obstetric warning system (MEOWS chart) as a predictor of obstetric morbidity: a prospective observational study. Eur J Obstet Gynecol Reprod Biol.

[26] Paternina-Caicedo A, Miranda J, Bourjeily G (2017). Performance of the Obstetric Early Warning Score in critically ill patients for the prediction of maternal death. Am J Obstet Gynecol.

[27] Blumenthal EA, Hooshvar N, McQuade M, McNulty J (2019). A Validation Study of Maternal Early Warning Systems: A Retrospective Cohort Study. Am J Perinatol.

[28] Henriksen L, Lukasse M (2016). Burnout among Norwegian midwives and the contribution of personal and work-related factors: A cross-sectional study. Sex Reprod Healthc.

