# Supplementary material for: Midwives’ experiences of using the Obstetric Norwegian Early Warning System (ONEWS): A national cross-sectional study
Source: Eur J Midwifery. 2021 Apr 22;5:10. doi: 10.18332/ejm/134510 (PMC8059414; doi:10.18332/ejm/134510)
Supplement: Supplementary file 1 [file EJM-5-10-s1.pdf]

Questionnaire – only questions not introduction

* = Obligatory questions
○ Only one box can be ticked off
☐ More than one box can be ticked off

This first part is about you as a midwife

How old are you? *

Answering options a «roller menu» with options from 25 to 70 *(midwifery is a masters (previously a diploma) education of 2 years after a bachelor in nursing with a required one years practice in between, thus the minimum age is 25)*

How long have you worked as a midwife? *

Answering options 0-4 years, 5-9 years, 10-14 years, 15-20 years, etc…with increasing time blocks of 5 years with the maximum experience being over 40 years.

Tick off for the type of midwifery education you have*

- ○ Diploma in midwifery from Norway
- ○ Masters in midwifery from Norway
- ○ Midwifery education from another country than Norway

Which Health authority (HF Helse Foretak) region do you work as a midwife? *

- ○ Health authority region West
- ○ Health authority region Central
- ○ Health authority region North
- ○ Health authority region South-East

What kind of maternity unit do you work at, as a midwife? *

If you work at an out-patient department or observation unit with acute admissions, or post-partum care at a hospital hotel, chose the unit your unit is linked with.

- Obstetric Unit (high-risk)
- Maternity ward (lower-risk)
- Labour room (midwife-led in small hospital)
- Midwife-led unit (can be free-standing or alongside)
- Other, specify*
  Possibility for free text

How many births are there a year at your maternity unit where you work as a midwife? *

- Less than 500
- 500-999
- 1000-1999
- 2000-2999
- More than 3000

This second part is about the use of the Obstetric Norwegian Early Warning Score (ONEWS)

How long has ONEWS been implemented/in use, there you work as a midwife? *

- 1-5 months
- 6-12 months
- 1-2 years
- Over 2 years
- Do not know

How long have you worked with ONEWS? *

- 1-5 months

- ○ 6-12 months
- ○ 1-2 years
- ○ More than 2 years
- ○ I do not know

What kind of course/instructions were you given in the use of ONEWS? *

- ☐ Course
- ☐ Study day/within unit teaching
- ☐ E-learing
- ☐ Taught by a colleague
- ☐ Self-study
- ☐ Nothing
- ☐ Other, please specify*

  **Possibility for free text*

Have you had adequate teaching/instructions to be able to monitor women with the use of ONEWS? *

- ○ Yes, I have received adequate teaching
- ○ No, I have not received adequate teaching
- ○ No opinion

Which women are being monitored using ONEWS at the maternity unit where you work as a midwife? *

- ○ Obstetric patients, from confirmed pregnancy until 6 weeks post-partum, but not during active labour
- ○ Obstetric patients, from confirmed pregnancy until 6 weeks post-partum, also during active labour

○ Do not know

Which criteria are used to monitor women with ONEWS during pregnancy at your maternity unit? *

- ☐ All women in outpatient consultations
- ☐ Only women with high-risk pregnancy in outpatient consultations
- ☐ All women admitted until discharge or birth
- ☐ All women admitted for observation, discontinued when the parameters are normal
- ☐ Only women with high-risk pregnancy admitted for observation, until discharge or birth
- ☐ Don’t know
- ☐ Other, please specify*

  [*]*Possibility for free text*

Which criteria are used to monitor women with ONEWS after a vaginal birth at your maternity unit? *

- ☐ All women within 2–3 hours postpartum
- ☐ All women within 2–3 hours postpartum, further monitoring discontinued when parameters are normal
- ☐ Only women classified as high-risk, within 2–3 hours postpartum
- ☐ Don’t know
- ☐ Other, please specify*

  [*]*Possibility for free text*

Which criteria are used to monitor women with ONEWS after Caesarian Section at your maternity unit? *

- ☐ All women after a CS and until discharge
- ☐ All women after a CS, further monitoring discontinued when parameters are normal
- ☐ Don’t know

☐ Other, please specify*

Possibility for free text

Which criteria are used to monitor women with ONEWS at the postnatal ward at your maternity unit? *

☐ All women with early discharge, 4–24 hours after birth

☐ All women in the postnatal ward, until discharge

☐ Only women in the postnatal ward who are classified as high-risk

☐ Only women in the postnatal ward who are classified as high-risk, discontinued when parameters normal

☐ Don't know

☐ Other, please specify

Possibility for fri text

Which criteria are used to monitor women with ONEWS upon readmission? *

☐ All women readmitted within 6 weeks postpartum until discharge

☐ All women readmitted within 6 weeks postpartum, discontinued when parameters normal

☐ Women only on indication

☐ Don't know

☐ Other, please specify

Possibility for free text

How often to you score women using ONEWS at the maternity unit where you work? *

☐ Once a day

☐ Twice a day

☐ Once a shift (8 hour shifts in Norway)

- ☐ On indication only
- ☐ Other, please specify*

  **Possibility for free text*

Please tick off all the items included in the ONEWS procedure at your unit *

- ☐ Respiration frequency
- ☐ Saturation (SpO2)
- ☐ Temperature
- ☐ Pulse
- ☐ Blood pressure
- ☐ Level of consciousness
- ☐ Total yellow score
- ☐ Total red score
- ☐ Urine (protein, glucose, other)
- ☐ Pain score 0-10
- ☐ Doctor contacted
- ☐ Other, please specify*

  **Possibility for free text*

Are there other observations you mean should be included in ONEWS? *

- ☐ Capillary pressure
- ☐ Status of the uterus (placement and contraction)
- ☐ Vaginal bleeding
- ☐ Urine (protein, glucose, other)
- ☐ Urinry retention
- ☐ Pain score 0-10
- ☐ Midwife concerned for the woman

☐ Doctor informed

☐ Doctor seen the woman

☐ Other, please specify*

Possibility for free text

Do you think the vital parameter values are adapted to women in the target group? *

☐ Yes

☐ No, please specify*

☐ Do not know

Possibility for free text

Third part concerns your own clinical experience with ONEWS

Please provide you opinion concerning the statements related to ONEWS*

	Agree totally	Agree partly	Neither agree nor disagree	Disagree partly	Disagree totally
'I see a clear purpose with the use of ONEWS'					
'I believe that ONEWS helps improve our knowledge about monitoring of vital parameters'					
'In my experience, ONEWS is often deprioritised in favour of other tasks that are considered more clinically relevant'					
'I believe that monitoring with ONEWS needs to be better adapted to each individual woman in the unit'					
'I believe that ONEWS helps improve patient safety'					
'I feel that monitoring with ONEWS is time-consuming'					
'I believe that ONEWS leads to better procedures for systematic monitoring'					
'I believe that monitoring with ONEWS is intrusive for the women, because of all the interruptions it involves'					
'I feel that it is challenging to defend monitoring with ONEWS to women who are not at any risk'					
'In my experience, ONEWS helps to better reveal early signs of illness'					

'I consider monitoring with ONEWS to be important when the woman is classified high-risk or shows signs of illness'	
'I find that ONEWS causes an increased medicalisation of normal pregnancy, perinatal and postnatal care practices'	
'In my contacts with the obstetrician, I feel that it is easier to be heard if I can refer to an ONEWS score'	
'I believe that ONEWS is a threat to the midwife's clinical judgement'	

Have you any other experiences concerning ONEWS which you consider relevant for this study? *

- ○ Yes, please tell us*
- ○ No
- ○ I do not know

Possibility for free text

Thank you very much for your contribution!

Vedlegg 3: Spørreskjema

SPØRREUNDERSØKELSE, kun spørsmål ikke intro

Basert på tekniske utfordringer med nedlasting av orginalversjon av spørreskjemaet fra nettskjema.no, er det her utarbeidet en identisk kopi av skjemaet.

* = Obligatoriske spørsmål

○ Kun et svar mulig

☐ Flervalg

Første del omhandler deg som jordmor

Hva er din alder? *

Svaralternativ i nedtrekks meny med alder under 25 år til over 70 år

Hvor lenge har du jobbet som jordmor? *

Svaralternativer i kategorier fra 0-4 år, 5-9 år, 10-14 år osv. Øvre kategori satt til over 40 år

Kryss av for ditt utdanningsnivå*

○ Videreutdanning i jordmorfag i Norge

○ Masterutdanning i jordmorfag i Norge

○ Utdanning som jordmor gjennomført i et annet land enn Norge

Ved hvilket helseforetak (HF) jobber du som jordmor? *

○ Helse Vest HF

○ Helse Midt-Norge HF

○ Helse Nord HF

○ Helse Sør-Øst HF

Ved hva slags fødeenhet jobber du som jordmor? *

Jobber du ved poliklinikk og/eller observasjonspost med innleggelse for øyeblikkelig hjelp, eller barselhotell, velg fødeenhet ditt arbeidssted hører innunder

- ○ Kvinneklinikk
- ○ Fødeavdeling
- ○ Fødestue
- ○ Jordmorstyrt enhet
- ○ Annet, vennligst spesifiser*
  **Mulighet for fritekst*

Hvor mange fødsler er det pr. år ved fødeenheten der du jobber som jordmor? *

- ○ Under 500
- ○ 500-999
- ○ 1000-1999
- ○ 2000-2999
- ○ Over 3000

Andre del omhandler Obstetric Norwegian Early Warning Score (ONEWS)

Hvor lenge har ONEWS vært innført ved fødeenheten der du jobber som jordmor? *

- ○ 1-5 mnd.
- ○ 6-12 mnd.
- ○ 1-2 år
- ○ Over 2 år
- ○ Vet ikke

Hvor lenge har du jobbet med ONEWS? *

- ○ 1-5 mnd.

○ 6-12 mnd.

○ 1-2 år

○ Over 2 år

○ Vet ikke

Hva slags opplæring har du fått i bruk av ONEWS? *

☐ Kurs

☐ Fagdag/internundervisning

☐ E-læring

☐ Opplæring av kollega

☐ Egenstudier

☐ Ingen

☐ Annet, vennligst spesifiser*

**Mulighet for fritekst*

Har du fått tilstrekkelig opplæring for å kunne overvåke kvinner ved bruk av ONEWS? *

○ Ja, jeg har fått tilstrekkelig opplæring

○ Nei, jeg har ikke fått tilstrekkelig opplæring

○ Ingen mening

Hvilke kvinner overvåkes ved bruk av ONEWS ved fødeenheten der du jobber som jordmor? *

○ Obstetriske pasienter, fra erkjent graviditet til 6 uker post-partum, men ikke i aktiv fødsel

○ Obstetriske pasienter, fra erkjent graviditet til 6 uker post-partum, og i aktiv fødsel

○ Vet ikke

Etter hvilke kriterier overvåkes kvinnen med ONEWS i svangerskapet ved fødeenheten? *

☐ Alle kvinner til poliklinisk konsultasjon

☐ Kun kvinner med risiko-svangerskap til poliklinisk konsultasjon

☐ Alle kvinner innlagt til observasjon ved fødeenheten, frem til hjemreise eller fødsel

☐ Alle kvinner innlagt til observasjon ved fødeenheten, men avsluttes etter normale parametere

☐ Vet ikke

☐ Annet, vennligst spesifiser*

**Mulighet for fritekst*

Etter hvilke kriterier overvåkes kvinnene med ONEWS etter vaginal fødsel ved fødeenheten? *

☐ Alle kvinner 2-3 timer postpartum

☐ Alle kvinner 2-3 timer postpartum, ved normale parametere avsluttes ytterligere overvåkning

☐ Kun kvinner klassifisert som risiko-fødende innen 2-3 timer postpartum

☐ Vet ikke

☐ Annet, vennligst spesifiser*

**Mulighet for fritekst*

Etter hvilke kriterier overvåkes kvinnene med ONEWS etter keisersnitt ved fødeenheten? *

☐ Alle kvinner etter keisersnitt og frem til hjemreise

☐ Alle kvinner etter keisersnitt, ved normale parametere avsluttes overvåkning etter gitt antall dager

☐ Vet ikke

☐ Annet, vennligst spesifiser*

**Mulighet for fritekst*

Etter hvilke kriterier overvåkes kvinnene med ONEWS på barselavdelingen ved fødeenheten? *

☐ Alle kvinner med tidlig hjemreise, 4-24 timer etter fødsel

☐ Alle kvinner ved barselavdelingen frem til hjemreise

☐ Kun kvinner ved barselavdelingen klassifisert som risiko-fødende

☐ Kun kvinner ved barselavdelingen klassifisert som risiko-fødende, ved normale parametere avsluttes overvåkning

☐ Vet ikke

☐ Annet, vennligst spesifiser*
Mulighet for fritekst

Etter hvilke kriterier overvåkes kvinnene med ONEWS ved re-innleggelse inntil seks uker postpartum? *

☐ Alle kvinner re-innlagt innenfor 6 uker postpartum, frem til hjemreise

☐ Alle kvinner re-innlagt innenfor 6 uker postpartum, ved normale parametere avsluttes overvåkning

☐ Kvinner kun på indikasjon

☐ Vet ikke

☐ Annet, vennligst spesifiser*
Mulighet for fritekst

Hvor ofte scores kvinnene ved bruk av ONEWS ved fødeenheten der du jobber? *

☐ En gang pr dag

☐ To ganger pr dag

☐ En gang pr vakt

☐ Kun på indikasjon

☐ Annet, vennligst spesifiser*

**Mulighet for fritekst*

I henhold til deres ONEWS prosedyre, kryss av for hva som er inkludert i deres kurve under? *

☐ Respirasjons frekvens

☐ Saturasjon (SpO2)

☐ Temperatur

☐ Puls

☐ Blodtrykk

☐ Bevissthet (AVPU)

☐ Totalt gul score

☐ Total rød score

☐ Urin (protein, glukose, annet)

☐ Smerte score 0-10

☐ Lege kontaktet

☐ Annet, vennligst spesifiser*

**Mulighet for fritekst*

Er det andre observasjoner du mener bør inkluderes i ONEWS kurven? *

☐ Kapillærfylling

☐ Uterus stand

☐ Vaginalblødning

☐ Urin (protein, glukose, annet)

☐ Urinretensjon

☐ Smerte score 0-10

☐ Jordmor bekymret for kvinnen

☐ Lege varslet

☐ Lege tilsett kvinnen

☐ Annet, vennligst spesifiser*

**Mulighet for fritekst*

Mener du grenseverdiene for vitale parametere er tilpasset kvinnene i målgruppen? *

☐ Ja

☐ Nei, vennligst spesifiser

☐ Vet ikke

Tredje del omhandler din egen kliniske erfaring med ONEWS

Vennligst ta stilling til følgende påstander vedrørende ONEWS*

	Helt Enig	Delvis Enig	Verken Enig/uenig	Delvis Uenig	Helt enig
Jeg opplever en tydelig hensikt med bruk av ONEWS					
Jeg mener at ONEWS bidrar til økt kunnskap om overvåkning av vitale parametere					
Jeg erfarer at ONEWS ofte nedprioriteres til fordel for andre oppgaver som anses som mer klinisk relevant					
Jeg mener at overvåkning med ONEWS i større grad må tilpasses den enkelte kvinne ved fødeenheten					
Jeg mener at ONEWS medfører forbedret pasientsikkerhet					
Jeg opplever overvåkning med ONEWS som tidkrevende					
Jeg mener at ONEWS medfører bedre rutiner for systematisert overvåkning					

Jeg mener at overvåkning med ONEWS er forstyrrende for kvinnene som følge av mange avbrytelser	
Jeg opplever det utfordrende å skulle forsvare overvåkning med ONEWS overfor kvinner uten risiko	
Min erfaring er at ONEWS bidrar til å i større grad avdekke tidlig tegn på sykdom	
Jeg anser overvåkning med ONEWS som viktig der kvinnen er klassifisert som risiko-fødende eller viser tegn til sykdom	
Jeg mener at ONEWS medfører økt sykeliggjøring av den normale svangerskap-, fødsel- og svangerskapsomsorgen	
I kontakt med gynekolog opplever jeg det enklere å bli hørt hvis jeg viser til score med ONEWS	
Jeg mener at ONEWS er en trussel mot jordmors kliniske blikk	

Har du andre erfaringer vedrørende ONEWS som du mener er relevant for denne studien? *

- ○ Ja, vennligst utdyp*
- ○ Nei
- ○ Vet ikke

**Mulighet for fritekst*

Tusen takk for din besvarelse!

© 2021 Fladeby L. K. L. et al.
